# Antioxidant Roles of SGLT2 Inhibitors in the Kidney

**DOI:** 10.3390/biom12010143

**Published:** 2022-01-16

**Authors:** Carmen Llorens-Cebrià, Mireia Molina-Van den Bosch, Ander Vergara, Conxita Jacobs-Cachá, Maria José Soler

**Affiliations:** 1 Nephrology and Transplantation Research Group, Vall d’Hebron Institut de Recerca (VHIR), Vall d’Hebron Barcelona Hospital Campus, Vall d’Hebron Hospital Universitari, 08035 Barcelona, Spain; carmen.llorens@vhir.org (C.L.-C.); mireia.molina@vhir.org (M.M.-V.d.B.); vergara.ander@gmail.com (A.V.); 2 Redes de Investigación Cooperativa Orientadas a Resultados en Salud (RICORS), RD21/0005/0016, Instituto de Salud Carlos III, 28029 Madrid, Spain

**Keywords:** redox, diabetic kidney disease, oxidative stress, mitochondrial dysfunction, SGLT2

## Abstract

The reduction-oxidation (redox) system consists of the coupling and coordination of various electron gradients that are generated thanks to serial reduction-oxidation enzymatic reactions. These reactions happen in every cell and produce radical oxidants that can be mainly classified into reactive oxygen species (ROS) and reactive nitrogen species (RNS). ROS and RNS modulate cell-signaling pathways and cellular processes fundamental to normal cell function. However, overproduction of oxidative species can lead to oxidative stress (OS) that is pathological. Oxidative stress is a main contributor to diabetic kidney disease (DKD) onset. In the kidney, the proximal tubular cells require a high energy supply to reabsorb proteins, metabolites, ions, and water. In a diabetic milieu, glucose-induced toxicity promotes oxidative stress and mitochondrial dysfunction, impairing tubular function. Increased glucose level in urine and ROS enhance the activity of sodium/glucose co-transporter type 2 (SGLT2), which in turn exacerbates OS. SGLT2 inhibitors have demonstrated clear cardiovascular benefits in DKD which may be in part ascribed to the generation of a beneficial equilibrium between oxidant and antioxidant mechanisms.

## 1. Introduction: The Redox System

In general terms, the reduction-oxidation (redox) system consists of the coupling and coordination of various electron gradients that are generated by serial reduction-oxidation enzymatic reactions. These reduction-oxidation reactions happen in fundamental biological processes in every cell [1] and lead to the production of radical oxidants or free radicals [2]. Free radicals are in fact oxygen or nitrogen metabolites that contain an unpaired electron and are thus partially reduced [3]. These free radicals have strong oxidizing capacity and can be classified into reactive oxygen species (ROS) and reactive nitrogen species (RNS) [4] (Table 1).

**Figure 1:**
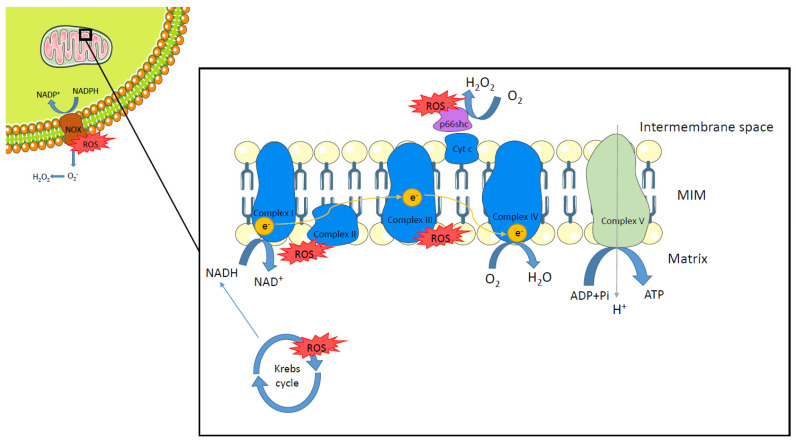
Sources of reactive oxygen species. Reactive oxygen species (ROS) are generated at different sites within the cell. Respiratory chain complexes I and III (in blue) produce ROS as a byproduct of electron transfer in mitochondria. Several enzymes involved in the Krebs cycle are also reported as sources of ROS. p66shc (in purple), by oxidizing cytochrome c (Cyt C), is able to generate ROS within the mitochondria intermembrane space. The NADPH oxidase (NOX) family of enzymes (in brown) are another important source of ROS by reducing oxygen to generate superoxide. MIM: mitochondria inner membrane.

The redox system is especially important during energy metabolism to generate adenosine triphosphate (ATP). The most important source of energy is glucose which is transformed into pyruvate via glycolysis. Pyruvate enters the tricarboxylic acid or Krebs cycle and reduces nicotinamide adenine dinucleotide (NAD) to NADH. Further, fatty acids (FA) can undergo β-oxidation to generate acetyl-CoA which then enters the Krebs cycle [5,6]. The NADH produced during the Krebs cycle is oxidized again in the mitochondrial respiratory chain [7]. The complexes that constitute the mitochondrial respiratory chain are NADH dehydrogenase (complex I), succinate dehydrogenase (complex II), cytochrome c reductase (complex III), cytochrome c oxidase (complex IV), and ATP synthase (complex V). These enzymes are able to oxidize NADH, starting the electron transport across the mitochondrial membrane and generating an electrochemical potential that allows complex V to generate ATP. Additionally, the electrons transported from complex I, through II and III, to complex IV are used to reduce oxygen to water [8,9]. ROS are generated as a byproduct of electron transfer. Therefore, mitochondria are considered the main source of ROS [10]. Approximately, 1–2% of the O_2_ consumed in the mitochondria is incompletely metabolized [11]. Here, the predominant origin of ROS is the respiratory chain (mainly complexes I and III), necessary for ATP synthesis. However, other mitochondrial enzymes have been reported to be sources of ROS, such as pyruvate dehydrogenase, α-ketoglutarate dehydrogenase, or succinate dehydrogenase, which are a part of the Krebs cycle [12]. Additionally, p66shc, an adaptor protein with proapoptotic activity, is involved in the production of ROS [13]. In vitro and in vivo experiments have shown that this protein can generate ROS by oxidizing cytochrome C [14,15]. The NADPH oxidase (NOX) family of enzymes are another source of ROS. NOX are multiunit enzymes that use NADPH as an electron donor to reduce oxygen, leading to the generation of superoxide. There are seven members of the NOX family, NOX1–5 and DUOX1 and 2. NOX enzymes are expressed in different tissues and differ in the domains that constitute them, for example, DUOX1 and 2 have an additional peroxidase domain at their N-terminal end [16,17] (Figure 1).

Regarding RNS, these comprise nitric oxide (NO) and its derivatives, such as peroxynitrite (ONOO^−^), dinitrogen trioxide (N_2_O_3_), dinitrogen tetraoxide (N_2_O_4_), nitrogen dioxide (NO_2_), or S-nitrosothiols (RSNO) [18,19] (Table 1). NO is produced from the metabolism of L-arginine [20]. The enzyme responsible for its synthesis is nitric oxide synthase (NOS) that converts L-arginine into L-citrulline, forming NO during the reaction. There are three forms of NOS: endothelial NOS (eNOS), neuronal NOS (nNOS) and inducible NOS (iNOS). eNOS and nNOS are constitutively expressed while iNOS is only expressed under specific stimuli like infection or trauma. The different NOS isoforms are expressed in different types of cells in which NO is involved in signaling and regulation of several cellular functions [21]. In mitochondria, NO reacts with complex III of the respiratory chain, inhibiting the electron transfer and enhancing the production of O_2_^−^. O_2_^−^ is highly reactive and may combine with NO, leading to the formation of peroxynitrite (ONOO^−^). ONOO^−^ is a powerful RNS that can irreversibly inhibit electron transport, which is pathological [18,22,23,24].

Both ROS and RNS are necessary for normal cell function as they modulate cell-signaling pathways and cellular processes. However, overproduction of any of them is pathological since it can damage macromolecules like proteins, lipids, or DNA [25]. DNA damage can lead to defective complexes I and III, which may result in a shutdown of mitochondrial energy production [26] and further increase ROS levels [27,28]. This eventually ends up causing mitochondrial damage and apoptosis [29,30]. Therefore, to regulate the generated ROS, there is a network of antioxidant systems to alleviate this stress. Superoxide dismutase (SOD) is an enzyme that contributes to the elimination of some oxidants. According to its subcellular location, we can differentiate between SOD1 (in the cytosol and mitochondrial intermembrane space), SOD2 (in the mitochondrial matrix), and SOD3 (in the extracellular matrix) [31]. SOD enzymes convert the superoxide radical into hydrogen peroxide [32,33] that is finally detoxified by catalase or glutathione peroxidase [34,35]. Catalases decompose H_2_O_2_ into water and oxygen, and glutathione peroxidases reduce H_2_O_2_ to water [36]. Glutathione has also a role in the antioxidant system, as it can either react directly with ROS and RNS or act as a cofactor for various enzymes helping the cell to maintain its redox status [37]. Further, exogenous and synthetic antioxidants can also be effective to prevent oxidative stress [38,39].

In this review, we will focus on oxidative stress-mediated mechanisms of kidney tubular dysfunction. Further, the contribution of the sodium/glucose co-transporter type 2 (SGLT2) in diabetic kidney disease (DKD) onset and the antioxidant potential of the SGLT2 inhibitors will be discussed.

## 2. Oxidative Stress-Induced Tubular Impairment

The tubular system has a very precise and highly regulated transport capacity between the tubule lumen and the bloodstream. A broad range of tubular transporters contribute to ion, amino acid, glucose, and other solute reabsorption. Therefore, tubular cells require a sufficient energy supply to sustain the high rate of exchange within the two compartments. Tubular cells are the most energy-demanding cells of the body after cardiac cells [40]. Their metabolism is highly dependent on oxygen consumption to produce ATP by oxidative phosphorylation. The oxidation of a glucose molecule provides a high energy yield (30–32 ATP) and is used as a substrate in every cell. However, in the kidney, each section of the tubule has its own preferences in terms of energy substrates, including amino acids, ketone bodies, or FA. Strikingly, FAs are an important energy fuel in tubular cells, providing 106–129 ATP through β-oxidation within the mitochondria [41]. Proximal tubular cells (PTC), located at the first section of the tubular system (segments S1, S2, and S3), are particularly energy-consuming, as 80% of the biomolecules filtered by the glomerulus are reabsorbed by these cells [42,43]. In PTC, ATP generation relies mostly on oxidative phosphorylation of FA rather than of glucose. However, under pathological conditions, such as acute kidney injury (AKI), which is associated with hypoxia [44], PTCs undergo a metabolic shift towards glycolysis [45].

As mentioned above, mitochondrial metabolism is a source of ROS that directly modulates cell-signaling pathways, protein function through post-translational modifications, and influences cell survival, proliferation, and apoptosis [46]. However, increased ROS production results in oxidative stress that causes damage to lipids, proteins, and nucleic acids, eventually disrupting cellular homeostasis. Overall, elevated ROS levels can lead to mitochondrial dysfunction, bioenergetics defects, altered gene expression, and cell death. PTCs have a high mitochondrial density that makes them susceptible to ROS-induced cell damage [42]. In vitro experiments performed using the HK-2 cell line (human PTCs) demonstrated an increase of oxidative stress, mitochondrial destabilization, DNA damage, apoptosis, and cell senescence when treated with hydrogen peroxide (H_2_O_2_) [47]. Aragno et al. showed that rats subjected to unilateral ischemia/reperfusion (I/R) injury exhibit high hydrogen peroxide levels in the cytosol obtained from kidney homogenate and high levels of nitrite/nitrate in serum compared to control and sham-operated animals. Consequently, the proximal tubules showed dilation and cell debris and cast formation in the lumen. These effects were attenuated in rats supplemented with dehydroepiandrosterone (DHEA), a steroid with antioxidant properties [48]. Beyond the respiratory chain, there are other sources of oxidants in the kidney. NOX enzymes have been recognized as a primary source of ROS in the kidney, especially NOX4. NOX4-derived H_2_O_2_ mediates several cell functions, but excessive levels can induce inflammation, apoptosis, fibrosis, and cell damage. NOX4 has constitutive activity, hence, the amount of H_2_O_2_ depends on the expression level. As a result, it can be damaging or beneficial depending on its abundance at the given time [49]. Normally, the levels of ROS increase in some AKI and chronic kidney disease (CKD) models, secondary to the overexpression of NOX4 [50]. Upregulation of renal NOX4 plays an important role in several pathologies, like DKD, hypertensive nephropathy, and polycystic kidney disease by increasing ROS levels and mitochondrial damage [51,52,53,54,55]. It has been described that mitochondrial ROS and NOX-produced ROS can enhance damage and depolarization of the mitochondrial membrane potential [46,56,57]. The total absence of NOX4 has also been reported to be pathological since it results in fibrosis and oxidative stress [58]. This could be in part related to the fact that H_2_O_2_ generated by NOX4 enhances nuclear factor erythroid 2-related factor 2 (Nrf2) stability, a regulator of antioxidant activity [59].

Excessive ROS production can damage mitochondria [60]. Mitochondrial damage has been recognized as a main contributor to tubular necrosis and apoptosis. Tubular cell necrosis involves disruption of respiration complexes, loss of mitochondrial membrane potential, and mitochondrial membrane transition, while apoptosis is caused by mitochondrial outer membrane permeabilization and release of apoptogenic factors [61]. Mitochondrial damage secondary to necrosis or apoptosis is especially problematic for the tubular cells as it may compromise its normal function and ultimately lead to renal dysfunction. Despite the effects of oxidative stress in kidney impairment having been studied extensively [42,46], its complexity and interconnection with a broad range of metabolic and cellular pathways make it puzzling to decipher its full implications in the onset, progression, and development of renal pathology.

## 3. Oxidative Stress in Diabetic Kidney Disease: Contribution of Sodium Glucose Co-Transporter Type 2 (SGLT2)

Albeit glucose consumption is low in the proximal tubule (except for segment S3 [62]), PTCs are primarily responsible for its reabsorption via the sodium/glucose co-transporter type 2 (SGLT2) [44]. SGLT2 is located at the brush border of PTCs in the S1 and S2 segments of the proximal tubule, where 90% of the total filtered glucose is reabsorbed [63]. Its transport is coupled with sodium (Na^+^/Glucose, 1:1) and it accounts for 5.7% of total renal Na^+^ reabsorption in healthy individuals [64]. Although SGLT2 activity has a role in maintaining the intrarenal osmolarity, the main sodium exchanger at the tubular level is the Na^+^/H^+^ Exchanger-3 (NHE3). Interestingly, NHE3 and SGLT2 have been shown to be interdependent [65]. Therefore, SGLT2 activity has a huge impact on both glucose and sodium balance. The Na^+^ gradient created by the sodium-potassium pump (Na^+^/K^+^-ATPase), located in the basolateral membrane of PTC, is harnessed by SGLT2 to transport glucose and sodium across the plasma membrane down-gradient [63]. At the same time, the basolateral glucose transporter, GLUT2, maintains the glucose gradient by passively easing its backflow into the bloodstream [66].

**Figure 2:**
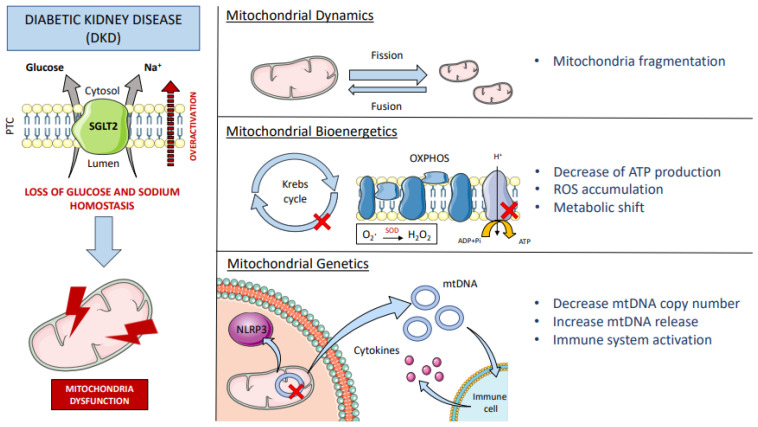
Mitochondrial dysfunction in DKD. The sodium/glucose co-transporter type 2 (SGLT2) is located in the apical membrane of the proximal tubular cells (PTC) of the kidney and is responsible for glucose and sodium uptake. Diabetic patients have overactivation of SGLT2 that alters glucose and sodium homeostasis, which directly affects mitochondrial function at different levels; imbalance of fission and fusion together with mitochondrial fragmentation (mitochondrial dynamics). Alteration and accumulation of Krebs cycle substrates, uncoupling of the oxidative phosphorylation chain (OXPHOS) with generation of ROS (e.g., formation of H_2_O_2_ by the enzyme superoxide dismutase (SOD)), leading to ATP depletion, oxidative stress, and a metabolic shift to oxygen-independent energy sources (mitochondrial bioenergetics). Mitochondrial DNA (mtDNA) is also damaged, leading to a reduction in mtDNA copy number and increased release of mtDNA into the cytosol, which activates the NLR family pyrin domain-containing 3 (NLRP3) inflammasome, and into the extracellular space, triggering the recruitment of immune cells and the onset of the inflammatory response (mitochondrial genetics). DKD: diabetic kidney disease; ROS: reactive oxygen species; ADP: adenosine diphosphate; ATP: adenosine triphosphate; NLR: NOD-like receptor.

SGLT2 plays a crucial role in glucose transport and intrarenal osmolarity. For that reason, the dysfunction of this co-transporter, which happens in diabetes, has a direct effect on intrarenal glucose metabolism and also on the redox environment. In diabetic patients, SGLT2 is overactivated, leading to increased glucose and sodium reabsorption [67]. The deleterious effects in kidney function and fluid homeostasis include the alteration of tubular-glomerular feedback (a self-regulated system of the kidney) and the increase of the glomerular filtration rate and the tubular reabsorption capacity. Systematically, the plasma volume and blood pressure increase, leading to hypertension, a common feature of these patients [68]. At the molecular level, this has drastic consequences for the activity of other transporters located in the tubule, such as NHE3, which is sensitive to small changes in sodium concentration. Packer et al. reviewed that Na^+^/H^+^ Exchanger-1 (NHE1) in the heart and vasculature and NHE3—the kidney isoform—are upregulated in heart failure and type 2 diabetes mellitus (T2DM) [69]. Additionally, when sodium levels drop in the luminal filtrate, Na^+^/K^+^-ATPase activity increases to restore the sodium gradient. As this transporter is ATP-dependent, its overactivation implies an increase in energy demand and oxygen consumption. It is worth mentioning that under normal conditions, 60% of kidney ATP consumption is intended for sodium uptake which is mostly attributed to basal Na^+^/K^+^-ATPase activity [70]. Therefore, the energy consumption of this transporter cannot be underestimated, and its impact on mitochondrial function should be further explored. The enhanced mitochondrial phosphorylation produces substantial accumulation of ROS products. In addition, it has been described that interleukin 6 (IL-6) activates SGLT2 through ROS in cultured tubular cells [71], which suggests that an inflammatory context would further promote ROS generation and SGLT2 overactivation. Thus, SGLT2 activity, oxidative stress, and inflammation actively contribute to the onset of DKD. By definition, DKD is a microvascular complication of diabetes mellitus that affects the kidney. These patients are diagnosed by the presence of persistent albuminuria, starting at first stage by hyperfiltration and ending with a progressive decrease in GFR at later stages. The Renal Pathology Society divide DKD pathology into glomerular, tubulointerstitial, and vascular lesions [72]. Focusing on the tubule, tubular membrane thickening, interstitial fibrosis, and tubular atrophy are usually observed. Coughlan et al. mapped the changes in mitochondrial adaptation in experimental rat’s kidney mitochondria at 4, 8, 16, and 32 weeks after induction of diabetes by streptozotocin intravenous injection [73]. The authors demonstrated that DKD begins with decreased ATP production in the renal cortex, mitochondrial fragmentation, which is accompanied by increased ROS, and early renal hyperfiltration. Their results suggest that mitochondrial dynamics and bioenergetic function worsen over time, preceding the development of albuminuria and histological lesions. An in vitro experiment with HK-2 cells found out that under high glucose conditions, HK-2 cells were susceptible to mitochondrial fragmentation. These effects were ameliorated with a SGLT2 inhibitor (empagliflozin), by regulating proteins of mitochondrial fission and fusion [74]. Mitochondrial fusion and fission are essential for mitochondrial maintenance but also for mitochondrial DNA (mtDNA) integrity, which can participate in the regulation of cell survival, metabolic processes, and redox-sensitive signals [75]. In addition, high glucose conditions increase superoxide formation in mitochondria, which combined with NO (released by endothelial cells under stress) generates peroxynitrite [76]. Peroxynitrite is a cytotoxic oxidant that induces eNOS uncoupling, restricting vascular relaxation, and promoting diabetic vascular complications. As mentioned before, NOX enzymes have been shown to mediate ROS production. NOX-derived ROS regulate many physiological mechanisms of the kidney, including tubular-glomerular feedback, glucose metabolism and transport, kidney hemodynamics, and electrolyte transport [77]. NOX4 is the major source of ROS production in podocytes, and it is upregulated under high-glucose conditions [78]. For this reason, NOX4 has been largely linked to DKD [53,79]. Induction of NOX4 in the Akita model of DKD results in the emergence of the typical structural changes seen in the diabetic kidney: glomerular hyperfiltration, mesangial matrix accumulation, glomerular membrane thickening, albuminuria, and podocyte loss [53]. In addition, metabolomic analysis performed on these mice revealed alterations in tubular energy metabolism with the accumulation Krebs cycle-related urinary metabolites [53]. The Krebs cycle is the central axis of energy production in mitochondria, thus, disproportion of the cycle substrates leads to mitochondrial dysfunction in advanced DKD [80]. Accordingly, a cross-sectional study in patients with and without DKD found that injured tubular cells showed several signs of mitochondrial damage, including (1) changes in mitochondrial dynamics, (2) decreased mtDNA copy number, and (3) increased release of mtDNA into the extracellular space [81]. Paradoxically, Cao et al. reported that plasma mtDNA content was decreased in patients with T2DM with clinically significant proteinuria (24 h urinary protein level >0.5 g) but not mild proteinuria (24 h urinary protein level ≤0.5 g), however, mtDNA abundance was increased in the urine of the former [82]. They propose that hyperglycemia reduces intracellular mtDNA and facilitates its extracellular release, so that circulating mtDNA may be filtered by the kidneys and end up in the urine [82]. In concordance, Kafaji et al. reported that a decreased mtDNA copy number in blood was associated with the severity and the presence of DKD [83]. Their group found out that mtDNA was lower in diabetic patients with macroalbuminuria than in patients with microalbuminuria or normalbuminuria. Therefore, altered mtDNA content can predict the occurrence of DKD and the oxidative stress environment [84]. Interestingly, release of mtDNA under cellular stress has been previously considered as a damage-associated molecular pattern (DAMP) [85]. mtDNA can be recognized by Toll-like receptor 9 (TLR9) and cystosolic Cgas-stimulator of interferon genes (STING) and activate the inflammasome, leading to kidney inflammation and fibrosis [86]. In this line, recent evidence showed an increase in macrophage infiltration in a mouse model of type 2 diabetes [87] and in human progressive DKD [88], which correlates with the disease state and renal injury. Other than the direct effect of mtDNA as DAMP, oxidative stress can induce inflammation in the kidney by stimulating cytokine production [89]. ROS derivates, which participate in cellular signaling, can activate the transcription factors nuclear factor kappa B (NFκB) and activator protein-1 (AP-1), promoting the transcription of cytokines, growth factors, and extracellular membrane proteins [89] (Figure 2).

Adding the immune response to the theme above reaffirms that mitochondrial dysfunction and redox imbalance lead to the emergence of the main precipitating factors: altered metabolism, oxidative stress, and inflammation. These three elements combine and reciprocally feed back into each other. As in the chicken-and-egg dilemma, determining which of these events triggers kidney disease in the first place entails a great difficulty. However, it is clear that pharmacological approaches focused on blocking/stimulating the involved pathways mentioned in this section and targeting the SGLT2 transporter are of particular interest.

## 4. Antioxidant Properties of SGLT2 Inhibitors

SGLT2 inhibitors (SGLT2is) are hypoglycemic drugs that target SGLT2 producing glycosuria. Consequently, SGLT2is decrease blood glucose in an insulin-independent manner, improving insulin resistance in diabetes [90,91]. To date, different SGLT2is, such as canagliflozin, dapagliflozin, ertugluflozin, and empagliflozin, have been approved to treat T2DM and DKD. Large clinical trials have demonstrated that these drugs (mainly canagliflozin, dapagliflozin, and empagliflozin) delay the progression of DKD on top of the standard of care with renin-angiotensin system (RAS) blockers [92,93,94,95]. The protective effect of the SGLT2is has been widely attributed to normalization of glycemia, natriuresis, body weight reduction, and decrease of intraglomerular pressure [63,96,97]. However, the use of SGLT2is has been associated with reduction of oxidative stress and inflammation as well (Table 2). In type 2 diabetic patients, twelve-week treatment with dapagliflozin significantly reduced the urinary excretion of 8-oxo-7,8-dihydro-2′-deoxyguanosine (8-oxo-dG), a marker of DNA oxidation [98]. In addition, empagliflozin treatment increased 2,2¢-azino-bis-(3-ethylbenzthiazoline-6-sulphonic acid) (ABTS) radical scavenging capacity (a measure of antioxidant capacity) in diabetic patients, suggesting a beneficial equilibrium between oxidant and antioxidant mechanisms despite empagliflozin also increasing serum levels of the thiobarbituric acid reactive substances (TBARS) and malondialdehyde (MDA). The authors suggest that the increased levels of TBARS and MDA (both indicators of lipid peroxidation) can be explained by the ketogenesis induced by SGLT2is that may contribute to lipid peroxidation [99]. Contrarily, Nabrdalik-Leśniak et al. have described that therapy of at least one month with either canagliflozin or empagliflozin decreased total antioxidant capacity (estimated by ABTS urine levels) in diabetic patients as compared to that of diabetic patients not treated with SGLT2is and healthy controls. However, the urinary activity of the antioxidant enzymes SOD and MnSOD was increased in diabetic patients treated with SGLT2is, suggesting that these drugs can also promote the activation of antioxidant mechanisms [100]. In this line, Iannantuoni et al. have described that 24-week long treatment with empagliflozin reduces superoxide production, increases glutathione content (an antioxidant), and enhances the expression of glutathione s-reductase and catalase in leukocytes of diabetic patients. In this study, the authors found that empagliflozin increased the serum levels of the anti-inflammatory interleukin 10 (IL-10) and decreased C reactive protein and myeloperoxidase serum levels suggesting, an antioxidant and anti-inflammatory role of SGLT2is [101]. Similarly, CD34+ endothelial progenitors (markers of endothelial function) isolated from peripheral blood of diabetic patients treated with canagliflozin showed increased SOD2, catalase, and glutathione peroxidase gene expression, which suggests that SGLT2is induce a beneficial antioxidant profile on these cells [102].

In diabetic experimental models, similar results have been found (Table 2). The administration of SGLT2is in rat and mice models decreases glycemia [106], and in experimental models of diabetic nephropathy, these drugs have demonstrated cardiorenal protection [103,107,108,109,110]. In a high-fat diet (HFD)-induced obesity mouse model, 8 weeks of empagliflozin administration improved cardiac dysfunction via Sestrin2-mediated AMPK-mammalian target of rapamycin (mTOR) pathways by maintaining the redox stability [104]. Similarly, canagliflozin administration for 12 weeks improved atherosclerosis lesions and endothelial dysfunction in streptozotocin-induced diabetic apolipoprotein E-deficient (ApoE−/−) mice. These beneficial effects could be in part ascribed to the reduction of oxidative stress, as canagliflozin decreased the expression of NOX2 and p22phox (both NADPH oxidase subunits) and the urinary excretion of 8-OHdG [105]. Regarding the kidney, Kamezaki et al. have demonstrated that ipragliflozin reduced renal NOX4 expression and oxidative stress in both the tubular epithelium and glomerular podocytes using a model of early diabetic nephropathy (db/db mice) [103]. In obese prediabetic rats, dapagliflozin suppressed renal gluconeogenesis and oxidative stress [111]. Thus, in a diabetic milieu, SGLT2is seem to promote antioxidant effects that could participate in the cardiorenal protective mechanisms of these drugs. Interestingly, blockade of SGLT2 (with SGLT2is or by silencing with siRNAs) ameliorates oxidative stress in endothelial [112,113], tubular [114,115,116], and mesangial cells [117] in culture. This suggests that these drugs have direct antioxidant effects on kidney cells and possibly on other organs (even off-target [113]).

The beneficial antioxidant effects of SGLT2is observed in DKD can in part be attributed to improvement of glycemia and blood pressure control, as other hypoglycemic drugs also reduce oxidative stress [98]. However, other protective mechanisms cannot be ruled out. Recent data obtained from EMPEROR-Reduced and DAPA-CKD studies have confirmed the efficacy of SGLT2is in patients with heart failure or CKD without a history of diabetes [118]. In the EMPEROR-Reduced double-blind trial, patients receiving empagliflozin had a lower risk of cardiovascular death or hospitalization and lesser GFR decline over time, regardless of the presence or absence of diabetes [119]. In addition, among CKD patients participating in the DAPA-CKD clinical trial, those treated with dapagliflozin had better stabilization of GFR and a lower risk of progression to end-stage renal disease or death, again, independent of the presence of diabetes [120]. Although the renal antioxidant effect of the SGLT2is in nondiabetic human CKD needs to be assessed in future research, studies in nondiabetic experimental models of kidney damage have already shown that these drugs protect the kidney by decreasing oxidative stress [121,122]. Furthermore, SGLT2is also have antioxidant properties in organs other than the kidney. Olgar et al. demonstrated that the increase of SGLT2 activity in cardiomyocytes of old Wistar male rats (24-month age) triggers ROS accumulation, alters mitochondrial dynamics, and promotes the loss of mitochondrial membrane potential. Interestingly, these cardiac events were mitigated by dapagliflozin treatment [123].

It has also been demonstrated that targeting SGLT2 with canagliflozin reduces cancer cell proliferation by inhibiting mitochondrial complex I-mediated respiration [124]. In the same line, dapagliflozin and canagliflozin produced cell growth arrest in breast cancer cells both in vitro and in vivo via the AMPK-mTOR pathway. The activation of the AMPK-mTOR pathway basically inhibits energy-consuming processes such as the mitochondrial phosphorylation and increases catabolic processes to restore cellular energy homeostasis [125]. Thus, as SGLT2is have antioxidant effects in nondiabetic CKD and other pathologies not related to the kidney, it is possible that the antioxidant properties of SGLT2is observed in DKD patients happen due to a combination of several factors: (1) glycemia and blood pressure control, (2) reduction of glucose concentration in the target cells, and (3) activation of pathways that improve glucose utilization. Further research is needed to ascertain the exact mechanism by which SGLT2is decrease ROS, but it is a fact that these drugs promote the restoration of the oxidant/antioxidant balance in the diabetic kidney as well as in other scenarios.

## 5. Conclusions

In conclusion, oxidative stress has an important role in DKD onset. High levels of glucose together with increased ROS production overactivate the SGLT2 transporter in tubular cells, which, in turn, exacerbates oxidative stress. The use of SGLT2is has demonstrated clear cardiovascular benefits which may be in part ascribed by a beneficial balance between oxidant and antioxidant pathways.

## Figures and Tables

**Table 1:** Main known reactive oxygen species (ROS) and reactive nitrogen species (RNS).

**Reactive Oxygen Species**	Superoxide	O_2_^−^
	Hydrogen peroxide	H_2_O_2_
	Hydroxyl radical	OH
**Reactive nitrogen species**	Nitric oxide	NO
	Peroxynitrite	ONOO^−^
	Dinitrogen trioxide	N_2_O_3_
	Dinitrogen tetraoxide	N_2_O_4_
	Nitrogen dioxide	NO_2_
	S-nitrosothiols	RSNO

**Table 2:** Summary of the antioxidant effects of SGLT2is observed in clinical and preclinical studies.

Publication	Study Design	SGLT2i Tested	Relevant Antioxidant Effects of the SGLT2i
van Bommel et al., 2020 [98]	**Clinical Trial:** Randomized, double-blind RED trial with 44 T2DM patients on metformin monotherapy (hemoglobin A1c 7.4%, mGFR 113 mL/min) treated for 12 weeks.	Dapagliflozin	Dapagliflozin reduced the urinary excretion of 8-oxo-7,8-dihydro-2′-deoxyguanosine (8-oxo-dG), a DNA oxidation marker.
Lambadiari et al., 2021 [99]	**Clinical Trial:** 160 T2DM patients with high cardiovascular risk (SCORE ≥ 10%). Follow-up duration was 12 months.	Empagliflozin	Empagliflozin treatment increased 2,2¢-azino-bis-(3-ethylbenzthiazoline-6-sulphonic acid) (ABTS) radical scavenging capacity, a measure of antioxidant capacity, and serum levels of the thiobarbituric acid reactive substances (TBARS) and malondialdehyde (MDA), indicators of lipid peroxidation.
Nabrdalik-Leśniak et al., 2021 [100]	**Clinical Trial:** Observational study of a total of 101 subjects, 33 healthy and 68 with T2DM, treated (37) or not (31) with an SGLT2 inhibitor for at least one month.	SGLT2 inhibitors (not specified)	SGLT2 inhibitors improve the superoxide dismutase (SOD) antioxidant defense.
Iannantuoni et al., 2019 [101]	**Clinical Trial:** Prospective follow-up study of 15 T2DM patients who received treatment with empagliflozin for 24 weeks.	Empagliflozin	Empagliflozin reduced superoxide production in leukocytes of diabetic patients and increased glutathione content, prominently after 24 weeks of empagliflozin treatment. Leukocyte expression of glutathione s-reductase and catalase, and serum levels of IL-10 were enhanced at 24 weeks of empagliflozin treatment.
Nandula et al., 2021 [102]	**Clinical Trial:** Double-blind, randomized trial of a total of 29 T2DM subjects (HbA1c of 7–10%) taking metformin and/or insulin were treated with canagliflozin or placebo for 16 weeks.	Canagliflozin	Canagliflozin increase the levels of the antioxidants superoxide dismutase 2 (SOD2), catalase and glutathione peroxidase in CD34+ vs cells of diabetic patients.
Kamezaki et al., 2018 [103]	**Preclinical study:** 8-weeks-old male type 2 diabetes (db/db) mice (*n* = 5), type 1 diabetes (BALB/c mice injected with STZ) mice (*n* = 5) and nondiabetic heterozygote (db/m) mice (*n* = 5) were administrated either ipragliflozin or placebo for 8 weeks.	Ipragliflozin	Ipragliflozin reduced cortical hypoxia and NADPH oxidase 4 expression, and subsequent oxidative stress, in early diabetic nephropathy.
Sun et al., 2020 [104]	**Preclinical study:** 6-week-old C57BL/6J mice and Sestrin 2 knockout mice were fed with normal chow diet or high-fat diet (HFD) for 12 weeks and then treated with or without empagliflozin for 8 weeks.	Empagliflozin	Empagliflozin reduced mitochondrial injury, and increased Sestrin 2 levels and AMPK and endothelial nitric oxide synthase phosphorylation, but inhibited Akt and mTOR phosphorylation. Additionally, empagliflozin enhanced the Nrf2/HO-1–mediated oxidative stress response.
Rahadian et al., 2020 [105]	**Preclinical study:** Male, 8-week-old ApoE−/− mice injected with STZ were treated with canagliflozin or placebo for 12 or 8 weeks.	Canagliflozin	Canagliflozin reduced the expressions of NADPH oxidase subunits (NOX2 and p22phox) and reduced urinary excretion of 8-oxo-dG.
